# On the Effect of Bimodal Rehabilitation in Asymmetric Hearing Loss

**DOI:** 10.3390/jcm10173927

**Published:** 2021-08-31

**Authors:** Simonetta Monini, Chiara Filippi, Alessandra De Luca, Gerardo Salerno, Maurizio Barbara

**Affiliations:** 1 ENT Unit, NESMOS Department, Sapienza University, 00189 Rome, Italy; simonetta.monini@uniroma1.it (S.M.); chiara.filippi@uniroma1.it (C.F.); al.deluca@uniroma1.it (A.D.L.); 2 Laboratory Unit, Sant’Andrea University Hospital, 00189 Rome, Italy; Gerardo.salerno@uniroma1.it

**Keywords:** bone conductive implant, asymmetric hearing loss, single sided deafness, hearing aid

## Abstract

Background: Bone conductive implants (BCI) have been reported to provide greater beneficial effects for the auditory and perceptual functions of the contralateral ear in patients presenting with asymmetric hearing loss (AHL) compared to those with single-sided deafness (SSD). The aim of the study was to assess the effects of wearing a conventional hearing aid in the contralateral ear on BCI in terms of an improved overall auditory performance. Methods: eleven AHL subjects wearing a BCI in their worse hearing ear underwent an auditory evaluation by pure tone and speech audiometry in free field. This study group was obtained by adding to the AHL patients those SSD subjects that, during the follow-up, showed deterioration of the hearing threshold of the contralateral ear, thus presenting with the features of AHL. Four different conditions were tested and compared: unaided, with BCI only, with contralateral hearing aid (CHA) only and with BCI combined with CHA. Results: all of the prosthetic conditions caused a significant improvement with respect to the unaided condition. When a CHA was adopted, its combination with the BCI showed significantly better auditory performances than those achieved with the BCI only. Conclusions: the present study suggests the beneficial role of a CHA in BCI-implanted AHL subjects in terms of overall auditory performance.

## 1. Introduction

Single-sided deafness (SSD) and asymmetric hearing loss (AHL) represent two distinctive audiological conditions that differ on the basis of having the contralateral, better-hearing ear either normal or impaired by hearing loss to different degrees. More specifically, AHL presents a pure tone average (PTA) of 70 dB or more in the worse ear and a PTA from 30 to 55 dB in the better ear, with an interaural threshold difference of least 15 dB [1,2,3]. The auditory rehabilitation of these conditions can be performed by either a contralateral route of signal (CROS) hearing aid, a bone conductive implant (BCI) or a cochlear implantation (CI), the latter only enabling near recovery to true binaural hearing.

Contrary to previous reports [4], it was recently shown that BCI implantation in the worse ear of AHL subjects could be beneficial for the auditory and perceptual functions, with better effects in the context of an SSD condition that, due to the presence of normal or near-normal function in one ear, obviously impedes any further hearing gain (ceiling effect) [5]. Furthermore, in both SSD and AHL conditions, an improved quality of the perceived sound along with quality-of-life parameters has also been reported [5,6,7,8,9]. 

It is a common observation in clinical practice that some subjects, first identified as SSD cases, show a deterioration of the hearing threshold in the better ear at a later stage, in relation to aging or to causing pathology or other. The temporal progression of the auditory function, in this case, could transform the features of SSD into those that characterize AHL, suggesting the adoption of a conventional hearing aid in the better, yet abnormal, hearing ear. 

By regularly monitoring SSD and AHL patients implanted with BCI in their worse ear, we observed that some of the SSD BCI-implanted subjects were undergoing an auditory deterioration in the contralateral ear that was consequently rehabilitated by a conventional hearing aid (CHA). 

The aim of the present study was to assess the efficacy of CHA in AHL BCI-implanted subjects, while comparing their audiological performances with those of unaided, BCI-only-aided and CHA-only-aided conditions.

## 2. Material and Methods

In total, 11 patients, 8 males and 3 females aged between 50 and 80 years old (mean 67.2), were included in the study for having previously received a BCI for SSD or AHL, according to the standardized classification [1,2]. The SSD etiology for undergoing BCI implantation and the presumed ones for requiring a CHA are shown in Table 1. All of the subjects have been monitored with follow ups ranging from 2 to 5 years (6 patients) and 5 to 10 years (5 patients). At the time of the present examination, all of the patients were classified as AHL since all of them showed moderate sensorineural or mixed hearing loss in the better ear, contralateral to the BCI implant. In view of this changed audiological situation, all of these patients were instructed to undergo a three-month trial with a retro-auricular, open-fitting CHA. After that period, they all underwent an audiological evaluation with pure tone audiometry to verify the 5-frequency pure tone average (250–4000 Hz), which was compared with that recorded at the end of the initial BCI procedure. Free-field pure tone and speech audiometry were also carried out under four conditions: unaided (unaided); only with a Baha processor on the worse ear, fitted as SSD (BAHA); only with a hearing aid on the better ear (CHA); and with a BAHA processor on the BCI and a contralateral hearing aid (BAHA + CHA). The free-field PTA, maximum word recognition score (WRS) and WRS in dB were consequently calculated under these four conditions. The absolute and mean values derived have been statistically analyzed. All of the patients signed an informed consent to participate in the clinical study, and the study was conducted according to the guidelines of the Declaration of Helsinki and approved by the Institutional Review Board of Sant’Andrea University Hospital. 

## 3. Statistical Analysis

The t-paired test was used to verify the hypothesis of difference among the groups, with a significant difference of *p* < 0.05.

## 4. Results

**Figure 1:**
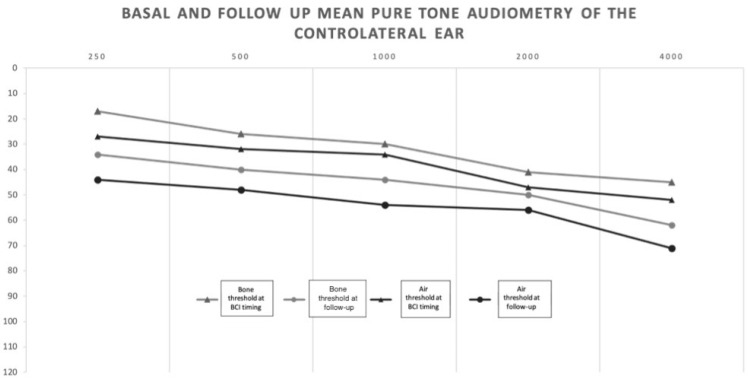
Mean basal air and bone conduction thresholds of the contralateral ear at BCI timing and at follow-up: transition from SSD to AHL.

The mean values of the air and bone conduction thresholds at the time of the implant (pre) and at the last follow-up (post) confirmed the hearing threshold deterioration in the better ear (Figure 1). The mean pre/post air PTA difference was 8.6 dB (min 0 dB–max 21 dB, SD 8.2). The mean pre/post bone PTA difference was 7.4 dB (min 0 dB, max 20 dB, SD 6.6).

**Figure 2:**
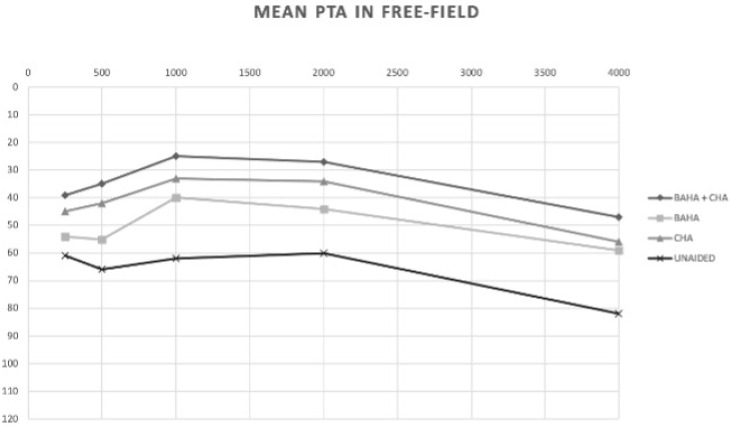
Comparison of free-field PTA in the unaided, BAHA, CHA and BAHA + CHA conditions.

The mean values of PTA, WRS maximum and dB HL related to WRS max in free field in the four different auditory conditions (unaided, CHA, BAHA and BAHA + CHA) are reported in Figure 2.

## 5. Mean Free-Field PTA Values

Unaided 61 dB HL (SD 16.5); CHA 41 dBHL (SD 7.4); BAHA 55 dBHL (SD 14.4); BAHA + CHA 37 dBHL (SD 7.6). The comparison of PTA under the different situations showed the following PTA gains: CHA vs. unaided 21 dB in favor of CHA (*p* = 0.0014); BAHA + CHA vs. unaided 26 dB in favor of BAHA + CHA (*p* = 0.0002); BAHA vs. unaided 6 dB in favor of BAHA (*p* = 0.03); BAHA vs. CHA 15 dB in favor of CHA (*p* = 0.0086); BAHA vs. CHA + BAHA 20 dB in favor to CHA + BAHA (*p* = 0.0008); CHA vs. CHA + BAHA 5 dB in favor of CHA + BAHA (*p* > 0.05) (Figure 1).

## 6. Mean Speech Audiometry Values in Quiet

Unaided: WRS 70% 74 dB; CHA: WRS 93% 60 dB; BAHA WRS 83% 65 dB; BAHA + CHA WRS 95% 56 dB.

**Figure 3:**
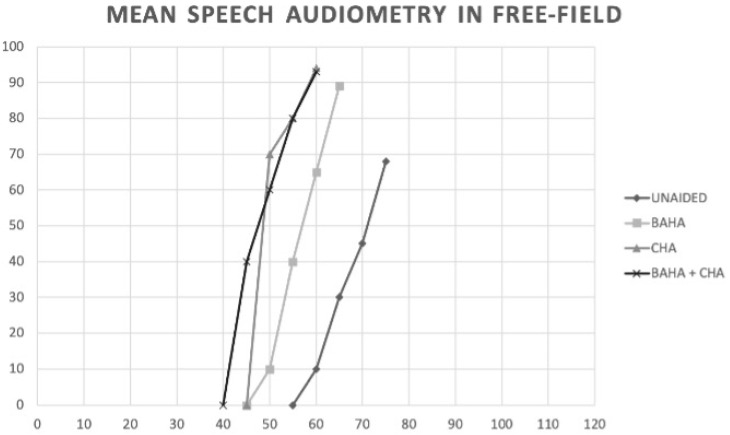
Comparison of % WRS and WRS dB HL in the unaided, BAHA, CHA and BAHA + CHA conditions.

The statistical paired analysis of the comparison among the different configurations showed the following differences: a significant improvement of 26% in WRS (<0.05) and of 14 dB HL in CHA vs. unaided (*p* = 0.0008); a significant improvement of 22% in WRS (<0.05) and of 9 dB HL in BAHA vs. unaided (*p* < 0.05); a significant improvement of 28% in WRS and of 18 dB HL in CHA + BAHA vs. unaided (*p* < 0.05); a non-significant improvement (*p* > 0.05) of 4% in WRS and of 5 dB HL in CHA vs. BAHA; a non-significant improvement (*p* > 0.05) of 2% in WRS and 4 dB HL in BAHA + CHA vs. CHA; a non-significant improvement of 6% in WRS and of 9 dB HL (*p* > 0.05) in BAHA + CHA vs. BAHA (Figure 3).

## 7. Discussion

The efficacy of BCI-mediated auditory rehabilitation in SSD and AHL is still controversial [1,4,5,6,7,10,11] given the evident differences between these two audiological conditions. It is well known that a BCI or CROS hearing aid for SSD could only partially restore some auditory cues that are lacking with the loss of binaural hearing; for example, with a greater improvement in the directional hearing of the implanted ear with respect to the contralateral one [8,12]. Moreover, when considering the effect of BCI on speech in noise in the context of SSD, the evidence is that the best effect is strictly correlated with the spatial separation of voice and noise, with the noise directed to the normal hearing ear and the speech to the implanted one, a situation that may not always be realistic in daily life [8,12].

Nevertheless, many implanting Centers, including ours, have collected over the last decade much clinical experience with BCI, both in SSD and in AHL. In this regard, a previous study reported the advantages of BCI in AHL patients, with a greater improvement of the auditory and perceptual functions with regard to the SSD condition, the advantage of this latter being attenuated by the ceiling effect due to the normal hearing function of the non-implanted ear [5]. The motivation of the present study is derived from this first evidence, with the aim of determining whether further audiological support, such as the one provided by a CHA on the better, yet impaired ear, could represent a substantially useful modality for increasing the subjects’ auditory performance. The primary goal of this study was to verify this hypothesis in a cohort of subjects that included AHL cases from the beginning along with SSD cases who displayed, at the time of the present examination, AHL features, owing to hearing deterioration in their better ears. Both sources of recruitment formed a homogeneous study group, as assessed when comparing in BCI-implanted SSD subjects the air and bone conductive thresholds at the times of the implant and at the last follow up, thereby motivating the inclusion of all of them as AHL.

The comparison of the four different conditions (unaided, contralateral CHA, BAHA and BAHA with CHA), provided some interesting results that are worth discussing. The “unaided” PTA and speech audiometry in quiet achieved similar results in the ear contralateral to the implant, whilst the comparison between the “unaided” condition and the other differently aided solutions resulted in significantly better scores for the latter. 

Considering the possibility of adopting only one solution, either a CHA or a BCI, it was found that CHA provided a better PTA threshold without similar significant advantages in WRS and loudness values. The possibility of implementing the auditory rehabilitation with more than one tool, as was tested in this study, shows that the combination of BAHA with CHA offers significant advantages in terms of PTA improvement compared to either BAHA or CHA only for PTA, WRS and loudness, but this latter finding was without statistical significance. These findings confirm the beneficial effects of a CHA in addition to a BCI for AHL patients, therefore verifying the study hypothesis of the positive role played by the CHA. 

The present study supports the findings of our previous study on the possibility of achieving, in AHL, the improvement of the auditory and perceptual functions of the contralateral better ear when the worse ear is implanted with a BCI. Moreover, it has allowed us to assess the effect of using a BCI to improve the outcome of wearing a CHA in the ear contralateral to the implant itself. It would therefore be advisable to perform a follow-up of both SSD and AHL patients rehabilitated with a BCI on the worse ear in order to monitor the auditory function of the better ear and eventually propose bimodal rehabilitation with a BCI and a CHA. 

## Figures and Tables

**Table 1:** Etiology of deafness in the SSD side that received a BCI and in the contralateral side, which showed deterioration over time.

ETIOLOGY OF DEAFNESS		
	BCI EAR	cHA EAR
1	TLB	PRESBYCUSIS
2	TLB	PRESBYCUSIS
3	SUDDEN DEAFNESS	UNKNOWN
4	SUDDEN DEAFNESS	PRESBYCUSIS
5	SUDDEN DEAFNESS	PRESBYCUSIS
6	UNKNOWN	PRESBYCUSIS
7	MUMPS	PRESBYCUSIS
8	TLB	PRESBYCUSIS
9	PETROSECTOMY	POST-TPL
10	TLB	PRESBYCUSIS
11	PETROSECTOMY	POST-TPL

## Data Availability

Not applicable.
